# High prevalence of *dhfr *triple mutant and correlation with high rates of sulphadoxine-pyrimethamine treatment failures *in vivo *in Gabonese children

**DOI:** 10.1186/1475-2875-10-123

**Published:** 2011-05-14

**Authors:** Ghyslain Mombo-Ngoma, Sunny Oyakhirome, Rosalynn Ord, Julian J Gabor, Katja C Greutélaers, Katharina Profanter, Benedikt Greutélaers, Florian Kurth, Bertrand Lell, Jürgen FJ Kun, Saadou Issifou, Cally Roper, Peter G Kremsner, Martin P Grobusch

**Affiliations:** 1 Medical Research Unit, Albert Schweitzer Hospital, Lambaréné, Gabon; 2 Institute of Tropical Medicine, University of Tübingen, Tübingen, Germany; 3 Département de Parasitologie, Université des Sciences de la Santé, Libreville, Gabon; 4 Medical Parasitology, New York University School of Medicine, New York, NY, USA; 5 London School of Hygiene & Tropical Medicine, Department of Infectious Tropical Diseases, London, UK; 6 Infectious Diseases, Department of Internal Medicine, Faculty of Health Sciences, University of the Witwatersrand, Johannesburg, South Africa; 7 Infectious Diseases, Tropical Medicine and AIDS, Division of Internal Medicine, Academic Medical Center, University of Amsterdam, The Netherlands; 8 Glaxo Smith Kline Biologicals, Rixensart, Belgium; 9 Department of Neonatology and Pediatric Intensive Care, University Hospital Carl Gustav Carus, Dresden, Germany; 10 Lindsay F. Kimball Research Institute, New York Blood Center, New York, NY, USA

## Abstract

**Background:**

Drug resistance contributes to the global malaria burden. *Plasmodium falciparum *dihydrofolate reductase (*dhfr*) and dihydropteroate synthase (*dhps*) polymorphisms confer resistance to sulphadoxine-pyrimethamine (SP).

**Methods:**

The study assessed the frequency of SP resistance-conferring polymorphisms in *Plasmodium falciparum*-positive samples from two clinical studies in Lambaréné. Their role on treatment responses and transmission potential was studied in an efficacy open-label clinical trial with a 28-day follow-up in 29 children under five with uncomplicated malaria.

**Results:**

SP was well tolerated by all subjects in vivo. Three subjects were excluded from per-protocol analysis. PCR-corrected, 12/26 (46%) achieved an adequate clinical and parasitological response, 13/26 (50%) were late parasitological failures, while 1/26 (4%) had an early treatment failure, resulting in early trial discontinuation. Of 106 isolates, 98 (92%) carried the triple mutant *dhfr *haplotype. Three point mutations were found in *dhps *in a variety of haplotypic configurations. The 437G + 540E double mutant allele was found for the first time in Gabon.

**Conclusions:**

There is a high prevalence of *dhfr *triple mutant with some *dhps *point mutations in Gabon, in line with treatment failures observed, and molecular markers of SP resistance should be closely monitored.

**Trial Registration:**

ClinicalTrials.gov: NCT00453856

## Background

*Plasmodium falciparum *malaria is a major cause of morbidity and mortality amongst children in sub-Saharan Africa; killing an estimated 1 million children below five years of age annually [1].

Chloroquine and sulphadoxine-pyrimethamine (SP) were, until fairly recently, the mainstays of malaria treatment in Africa. Resistance to chloroquine is now widespread, while resistance to SP increases. When used as first line treatment for malaria, SP exerts a strong selection pressure on parasite populations, thus increasing the frequency of resistance mutations in the *dihydrofolate reductase *(*dhfr*) gene which codes for the enzyme DHFR (the target of pyrimethamine) [2,3] and the *dihydropteroate synthase *(*dhps*) gene which codes for the enzyme DHPS (the target of sulphadoxine) [4,5] in the parasite reservoir [6]. These mutations are known to confer resistance to the component drugs of SP in *P. falciparum*, and this rise in resistance has been well documented in studies in vivo throughout the African continent [7-11]. Consequently, there is now a general move towards artemisinin-based combination therapy as first line anti-malarial treatment of uncomplicated *falciparum *malaria [12]. As elsewhere, in the Lambaréné area in the Moyen Ogoué Province, Gabon, artemisinin-based combination therapy is increasingly frequently prescribed and administered (Profanter et al., submitted for publication). However, the use of SP for curative treatment in Gabon is still common because it is affordable on household level. SP is also recommended for use as intermittent preventive treatment of malaria in pregnancy (IPTp) [13] whilst intermittent preventive treatment in infants (IPTi) with SP has been evaluated in detail [14] and has recently been adopted by the World Health Organization as an additional malaria control tool [15]. Given these facts, monitoring of SP resistance remains an important task.

Different combinations of point mutations in the parasite's *dhfr *and *dhps *genes confer varying levels of drug tolerance. A *dhfr *codon 108 exchange (Ser-108 to Asn-108) confers mild pyrimethamine tolerance. The additional presence of Ile-51 and Arg-59 confers pronounced resistance to pyrimethamine and predicts SP treatment failure in some areas. Similarly, for *dhps*, mutant Gly-437 is associated with some sulfonamide resistance while additional changes Glu-540, Gly-581, and Ser-613 appear to increase its degree [16]. A common mutation Ala-436 is generally considered an alternative wild type polymorphism [17]. The combination of the *dhfr *triple mutant together with a *dhps *Gly-437 + Glu-540 double mutant has been shown to predict SP treatment failure in East African settings [18,19] but this genotype is rare in West Africa.

This paper reports the prevalence of *dhfr *and *dhps *point mutations in *P. falciparum *isolates collected from children with malaria attacks in Lambaréné from 2005 to 2007, and the associations of these point mutations with SP treatment outcome.

## Methods

### Study area and samples

Blood samples positive with *P. falciparum *were collected from patients and asymptomatic individuals during two separate studies from 2005 to 2007, initiated by the IPTi consortium [14] and carried out at the Medical Research Unit of the Albert Schweitzer Hospital in Lambaréné, Gabon. In this study area of 30,000 inhabitants, malaria transmission is perennial, with little seasonal variation [20,21].

One study was an IPTi trial (ClinicalTrials.gov identifier: NCT00167843) reported elsewhere [21,22] and included into a meta-analysis of IPTi-SP trials across Africa [14].

The second study as reported here was an in vivo therapeutic efficacy trial of SP in children aged 6-59 months with uncomplicated *falciparum *malaria (ClinicalTrials.gov identifier: NCT00453856). Both studies were approved by the ethics committee of the International Foundation of the Albert Schweitzer Hospital.

Both studies included children from Lambaréné and its vicinity. Informed consent was obtained from parents or legal representative for each subject prior to enrolment. Children with acute febrile disease were physically examined; a thick blood film was screened for malaria parasites and a finger-prick blood sample for filter paper blood spotting (FTA Classic Card, Whatman Inc., Sanford, ME, USA) was obtained. The filter paper blood spots were air dried and stored at 4°C in individual plastic bags with desiccant and genotyping analysis later performed at the London School of Hygiene and Tropical Medicine (London, UK).

### SP treatment in vivo study-specific procedures

The target sample size of 139 children was determined from a therapeutic efficacy between 70 and 95%, with a precision of 7.5% allowing for a 20% drop-out. The study end points were adequate clinical and parasitological response, late clinical failure, late parasitological failure and early treatment failure as defined by the World Health Organization [23]. These were calculated in the per-protocol population, while safety and tolerance were evaluated in the intention-to-treat population, all children who received single-dose SP.

Study physicians examined all eligible children at enrolment, recording blood pressure, pulse and axillary temperature. Laboratory data recorded were parasite density of asexual and sexual forms of malaria, haemoglobin, haematocrit, white blood cell count, thrombocyte count (ABX Pentra 60^®^, ABX Diagnostics, Montpellier, France), creatinine and alanine- aminotransferase (ABX Mira Plus^®^, ABX Diagnostics).

A single dose of SP (25 mg/kg and 1.25 mg/kg; Maneesh Pharmaceuticals PVT Ltd, Govandi Mumbai, India) was crushed and mixed with glucose solution and administered orally by a study clinician. A patient was withdrawn from the trial if a re-dose was vomited and the outcome was early treatment failure, late clinical failure or late parasitological failure according to WHO definitions [23]. These patients were subsequently treated with oral artemether-lumefantrine (COARTEM^®^, Novartis Pharma Ltd Beijing for Novartis Pharma AG, Basle Switzerland) or hospitalized, if oral treatment was not tolerated. Scheduled follow up visits of all study subjects were on days 1, 2, 3, 7, 14, 21 and 28 after oral administration of SP (day 0).

### DNA extraction and PCR amplification of *dhfr *and *dhps *genes

DNA was extracted from bloodspots dried on filter papers by soaking overnight in 1 mL of 0.5% saponin-1x phosphate buffered saline. The segment was then washed twice in 1 mL of PBS and boiled for 8 min in 100 μL PCR quality water with 50 μL 20% Chelex suspension (pH 9.5).

*Dhfr *and *dhps *were PCR amplified using a nested PCR. The outer and nested *dhfr/dhps *PCR conditions, including primer sequences and reaction parameters, were as previously described [24]. The nested PCR products were confirmed by electrophoresis on a 1% agarose gel along with a set of controls.

### Molecular genotyping of point mutations using SSOP

Point mutations at codons 51, 59, 108 and 164 of the *dhfr *gene and codons 436, 437, 540, 581, and 613 of the *dhps *gene were genotyped using the Sequence Specific Oligonucleotide Probes (SSOP), a dot-blot methodology previously described by Pearce and colleagues [24]. The probed blots were visualized through alkaline phosphatase-catalyzed breakdown of the fluorogenic substrate (ECF) (GE Healthcare, Buckinghamshire, UK) and the chemifluorescent signal scanned on a TYPHOON Trio^® ^Phosphoimager (GE Healthcare, Buckinghamshire, UK).

The stringency and specificity of the hybridization process was confirmed by inspection of a series of four controls of known single genotype variant sequence. All blots with non-specifically bound probes were stripped and re-probed. A sequence variant was considered to be present in the PCR product when the intensity of signal was higher than that of the background. The presence, absence, and relative abundance of hybridization signal were recorded for every probe at each locus. Blood samples were categorized as having a single, a majority plus a minority, or a mixture of sequences at every locus. A sample was considered to have a single haplotype when only one sequence variant was found at each locus. Majority and mixed genotype infections were differentiated according to the relative intensity of signal.

### Molecular treatment outcome measures

Each *P. falciparum *infection was characterized on the basis of the *MSP-2 *polymorphism [25]. Allele-specific PCR to amplify *FC27 *or *IC1*/*3D7 *fragments was performed on paired pre- and post-treatment bloodspot samples. Cases in which pre- and post-treatment genotypes were identical were considered as recrudescence, i.e. failures; cases in which pre- and post-treatment genotypes were different were considered as re-infections; mixed genotypes were classified as failures. Parasite clearance time was defined as the time from starting SP treatment until parasites were undetectable in two consecutive peripheral blood films at least 24 hrs apart. An experienced laboratory technician measured asexual parasitaemia per μL according to the Lambaréné method [26]. The presence of single or multiple *dhfr *or *dhps *mutations from samples collected prior to treatment with SP were examined for their association with patients' treatment outcome. Each isolate was coded based on the presence or absence of a resistance associated allele. For example, infections with mixed wild-type/mutant alleles were treated as mutant.

Transmission potential was evaluated by measuring gametocytaemia on enrolment and at scheduled visits following the same technique used for asexual parasitaemia.

### Statistical methods

The study analysis consisted on calculating the proportion of malaria infections with mutations of interest present in samples from the IPTi plus those present at the time of enrolment in the SP in vivo study and estimated 95 percent binomial confidence intervals for prevalence of mutations in the study area.

For the SP treatment in vivo study, data were entered into an electronic database and validated by complete manual review. Statistical analysis was performed using Stata (Stat Corp., College Station, TX, USA) statistical software. Fever clearance time was calculated as the time from the start of treatment to the first of two consecutive axillary temperature measurements that recorded below 37.5°C and parasite elimination time as the time from the start of treatment to that of two consecutive negative blood smears.

## Results

### SP in vivo treatment response

**Figure 1:**
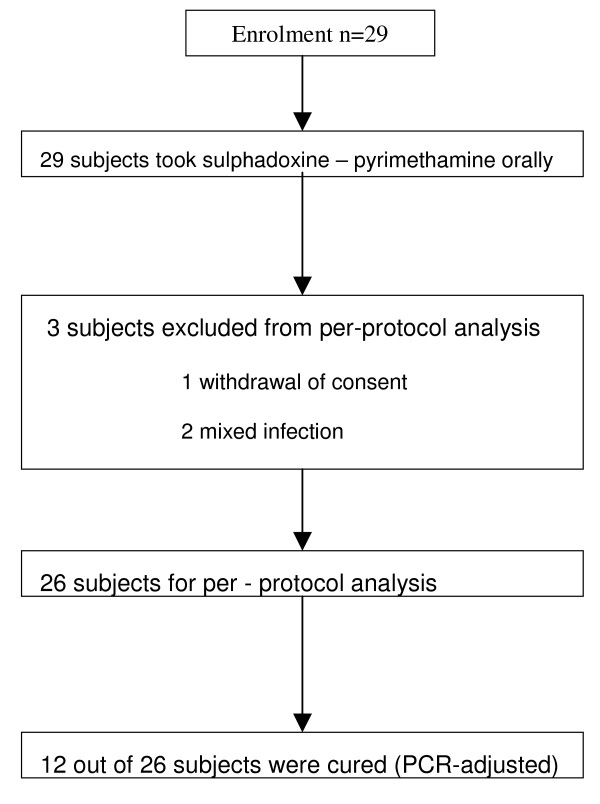
**Study flow chart**.

Of the 29 patients who received a standard oral dose of SP in the study assessing the in vivo efficacy of SP, 18 (62.1%) completed the 28-day active follow-up, before the trial was stopped for safety concerns. The targeted sample size was not attained because after 29 subjects were enrolled, a decision to stop the trial was taken by the investigators because of concerns about the early treatment failure. Figure 1 provides the study flow, and Table 1 provides baseline characteristics of patients and details on day 28 cure rates. Clinically, 14 subjects developed treatment failure, with one early and 13 late treatment failures. Three patients were excluded from per protocol analysis, because of mixed infections with other *Plasmodium *species (n = 2), and parents withdrawal of a consent for one child at the follow-up visit day 28. Matched sample pairs collected before and after treatment from 26 subjects were analyzed at the *msp-2 *locus. Overall, 12 subjects (46%) had an adequate clinical and parasitological response, including two subjects with parasites on day 28 post-treatment which were new infections, while 14 (54%) carrying parasites in the post-treatment sample probably failed SP treatment.

**Table 1:** Baseline Characteristics and results of the SP efficacy study

Number of patients	29
**Age, mean (SD), months**	34.8 (16.6)

**Sex ratio (male/female)**	1.9 (19/10)

**Weight, mean (SD), kg**	13.0 (2.5)

**Axillary temperature at admission, mean (SD), °C**	37.1 (0.95)

**Parasitaemia at admission, median (quartiles)**	18,718 (2,800-33,300)

**Day 28 PCR adjusted cure in PP analysis, n (%) Day 28 unadjusted cure in PP analysis, n (%)**	12/26 (46) 10/26 (38.5)

**Early treatment failures, n (%)**	1 (4)

**Late parasitological failures, n (%)**	13 (50)

**Parasite clearance time, mean (95%CI), hours**	61 (45-76)

**Fever clearance time, mean (95%CI), hours**	22 (14-30)

**Gametocyte carrier rate at admission, n (%)**	2 (6)

**Gametocytes carrier rate during follow-up, n (%)**	22 (85)

SD: Standard Deviation, PCR: Polymerase Chain Reaction, CI: Confidence Interval, n: number of patients.

Mean fever clearance time was 22 hours, parasite elimination time was 61 hours and the gametocytes carriage rate was 85%. On enrolment (D0) the mean haemoglobin was 9.4 g/dL; 45% of the subjects (13/29) had a haemoglobin less than 9.0 g/dL - a mean change in hemoglobin between D0 to D28 of 1.5 g/dL (CI; 1 - 2.1, p < 0.0001).

Oral SP was well tolerated by all 29 subjects. There were 26 adverse events reported during the course of this study; worm infestation (n = 9), respiratory infection (n = 8), anaemia (n = 4), skin infections (n = 4) and diarrhoea (n = 1). There was no serious adverse event observed during this trial except the hospitalization (per protocol) of one patient who had an early treatment failure.

### Prevalence of *dhfr *and *dhps *point mutations

Of the total 155 *falciparum*-positive samples collected in Lambaréné from 2005 to 2007 and sent for analysis, 106 yielded PCR products for *dhfr *and 121 for *dhps*. Among these, 5% were infections with mixed strains at *dhfr *and 26% were mixed at *dhps*. The high number of mixed infections was a reflection of the high level of transmission in the area.

Three point mutations were found in *dhfr; *51I, 59R and 108N, while 164L and 108Thr were absent. A high proportion of infections (98/106; 92%) were found to have the triple mutant *dhfr *51I-59R-108N, while the sensitive wild-type was absent. The single mutant 59R (NRS) was present only in one sample (1%) (Table 2). Three point mutations were found in *dhps*; 436A, 437G and 540E, while 581G and 613T were absent. The prevalence of *dhps *single mutant haplotypes containing 436A alone, 18/121 (15%) or 437G alone, 60/121 (50%) were higher than double mutant haplotypes *dhps *436A + 437G, 6/121 (5%) and 437G + 540E, 4/121 (3%) (Table 2). The *dhps *wildtype haplotype (SAK) was found in 2/121 (2%) of infections.

**Table 2:** Prevalence of *dhfr *and *dhps *haplotypes in isolates from *Plasmodium falciparum *positive patients from Lambaréné, Gabon, 2005-2007

*dhfr*	*N = 106*	*%*
**Single mutant**	1	1

**Double mutant**	2	2

**Triple mutant**	98	92

**MIX**	5	5

***dhps***	***N = 121***	***%***

**Sensitive**	2	2

**Single mutant (436A only)**	18	15

**Single mutant (437G only)**	60	50

**Double mutant (436A + 437G)**	6	5

**Double mutant (437G + 540E)**	4	3

**MIX**	31	26

MIX: infections with mixed strains

The combined *dhfr *and *dhps *haplotypes were available for 102 samples. The triple mutant *dhfr *51I-59R-108N was found in combination with *dhps *437G in 44 isolates (43%). Of these, just 4 (4%) carried the 'quintuple' genotype (*dhfr *51I-59R-108N + *dhps *437G + 540E), which is predictive of SP treatment failure. The combined sensitive alleles for *dhfr *and *dhps *were not observed in any of the isolates. Therefore, *dhfr/dhps *combined individual infections according to the number of mutations at *dhfr *and *dhps *codons were categorized as M3, M4, and M5 (Table 3).

**Table 3:** Combined *dhfr *and *dhps *haplotypes

*Combined dhfr and dhps haplotypes*			N = 102	*%*
*dhfr*_**51I**-59C-**108N **+ *dhps*_**436A**-437A-540K	ICN + AAK =	M3	2	2

*dhfr*_**51I**-**59R**-**108N **+ *dhps*_436S-437A-540K	IRN + SAK =	M3	2	2

*dhfr*_**51I**-**59R**-**108N **+ *dhps*_**436A**-437A-540K	IRN + AAK =	M4	16	16

*dhfr*_**51I**-**59R**-**108N **+ *dhps*_436S-**437G**-540K	IRN + SGK =	M4	44	43

*dhfr*_**51I**-**59R**-**108N **+ *dhps*_**436A**-**437G**-540K	IRN + AGK =	M5	4	4

*dhfr*_**51I**-**59R**-**108N **+ *dhps*_436S-**437G**-**540E**	IRN + SGE =	M5	4	4

*dhfr*_**51I**-**59R**-**108N **+ *dhps*_MIX	MIX		24	24

*dhfr*_51N-**59R**-108S + *dhps*_MIX	MIX		1	1

*dhfr*_MIX + *dhps*_436S-**437G**-540K	MIX		1	1

*dhfr*_MIX + *dhps*_MIX	MIX		4	4

Of the 29 pre-treatment isolates, the analysis of the *dhfr *product at each of the codons found with point mutations - 51, 59 and 108 - showed that most of the infections were from mutant strains, as depicted in Table 4. Interestingly, all the positive samples at post-treatment carried the point mutations 51I (8/26), 59R (12/26), and 108N (10/26) (Table 4).

**Table 4:** Prevalence of *dhfr *gene mutations at enrolment and at day of failure in children included in the in vivo study

*DHFR*				
**Pre-treatment**	**N = 29**			

*Codon*	*51*	*59*	*108*	*164*

**Sensitive**	**N**	**C**	**S**	**I**

	1 (3%)	2 (7%)	0	28 (97%)

**Mutant**	**I**	**R**	**N**	**L**

	23 (79%)	26 (90%)	26 (90%)	0

**No PCR product**	5	1	3	1

**Post-treatment**	**N = 26**			

*Codon*	*51*	*59*	*108*	*164*

**Sensitive**	**N**	**C**	**S**	**I**

	0	0	0	12 (46%)

**Mutant**	**I**	**R**	**N**	**L**

	8 (31%)	12 (46%)	10 (38%)	0

**No PCR product**	18	14	16	14

Regarding *dhps*, an analysis specific to the combined codons 436/437 showed prior to treatment that the 436/437SG was the most frequent haplotype either in mono- or mixed infections and also post-treatment (Table 5).

**Table 5:** Prevalence of *dhps *gene mutations at enrolment and at day of failure in children included in the in vivo study

*DHPS*					
**Pre-treatment**					

*436/437 SA*	*436/437 SG*	*436/437 AA*	*436/437 AG*	N = 29	%

0	1	0	0	11	38

1	0	0	0	1	3

1	1	0	0	1	3

0	1	0	1	2	7

0	0	1	0	2	7

0	1	1	0	7	24

1	0	1	0	1	3

0	1	1	1	4	14

**Post-treatment**					

*436/437 SA*	*436/437 SG*	*436/437 AA*	*436/437 AG*	N = 26	%

0	0	0	0	11	42

0	1	0	0	10	38

1	1	0	0	1	4

0	0	0	1	1	4

0	0	1	0	2	8

0	1	1	0	1	4

SA: sensitive, wild-type; SG: single mutant 437G; AA: single mutant 436A; double mutant 436A + 437G

### Mutations and parasite clearance time

The cumulative parasite clearance time distribution for the 29 study subjects was as follows: 17% (5/29), 57% (13/23) and 60% (6/10) cleared parasites by days 1, 2 and 3, respectively. Subjects with a parasite clearance time longer than 3 days (n = 4), included two children with treatment failure and two children with adequate responses. Two subjects (8%) did not clear parasites until the visit after day 7 of treatment and were considered as late parasitological failure. The proportion of subjects who by Day 3 after treatment have not cleared parasites (~40%) was relatively high and certainly associated with the mere presence of the *dhfr *triple mutant, as the risk estimate did not increase when comparing *dhfr *triple mutant alone and combined with *dhps *single and double mutants, odds ratio (OR) (95% CI) for parasite clearance time longer than 3 days of 1.9 (0.1 - 52.8) and 1.3 (0.03 - 53.6), respectively.

### Mutations and treatment outcome

Drug and iron plasma concentrations, or biostatistics information like age and sex were not taken into account, as any influence on treatment response has not been reported previously [27]. The very small number of patients does not allow for strong conclusions; however, the high rates of SP treatment failure in this study obviously correlate with the high rates of *dhfr *triple mutant isolates, and with the emergence of *dhps *540E under treatment. The addition of *dhps *mutations had not increased this risk, as OR (95% CI) for *dhfr/dhps *combined were 6.3 (0.2 - 180.4) and 3.0 (0.1 - 115.4), respectively, for M4 and M5.

Peak gametocytaemia occurred 7 days after treatment, when 17 (50%) subjects had detectable gametocytes, up from 2 (6%) at baseline. *Dhps *haplotypes were not associated with gametocyte prevalence or density at any time.

## Discussion

These findings show a high prevalence (98/106; 92%) of parasites with triple mutant 51I-59R-108N *dhfr *haplotype in the study area. Previous studies in Gabon point to an increase in the prevalence of resistant *dhfr *in recent years. Fifty percent prevalence of 108N was found in Lambaréné in 1996 [28], and 90% was reported from Franceville in 1998 [29]. A later study conducted in Bakoumba in 2000 [30] reported a prevalence rate of 72% of triple-mutant *dhfr*. Consistent with those earlier studies no mutation at *dhfr *codons 164 (Leu-164) was found in the study reported here; neither Thr-108, which is associated with cycloguanil resistance [28-30].

In the in vivo study assessing the SP efficacy, the results show an unacceptable PCR-corrected failure rate of 54%. In earlier studies assessing the in vivo efficacy of SP in Gabon from 1995 - 2006 cure rates were higher, ranging from 69% to 95% [10,27-34]. Similarly, in vitro studies also showed an increasing rate of resistance, from 30% in 1992 [35] to 75% in 2000 [30], which seems to correlate with the increase of the *dhfr-*resistant genotypes as well. At the *dhps *codons, the most frequent haplotypes were single mutant *dhps *haplotypes 436A and 437G (Table 2). The prevalence of 437G overall was 58% (Table 2), which compares to 28% (10 of 36) in Lambaréné in 1996 [28], 37% (14 of 38) in Franceville in 1998, [29] and 63% (70 of 110) in Bakoumba in 2000 [30].

Because the clinical trial had to be terminated prematurely on the grounds of unexpected high therapeutic failure rates, it was underpowered to show a statistical correlation between the presence of *dhfr or dhps *mutation and SP efficacy. The finding of high prevalence of *dhfr *triple mutant and *dhps *437G mutations, coincidental with high rates of failures point to deterioration of SP treatment efficacy in Gabon. These findings suggest that in Gabon, the prevalence of the *dhfr *triple mutation and *dhps *437G and 540E could be used as tool to screen for and monitor SP resistance. Although factors associated with treatment success such as drug absorption, immunity and folate levels [36,37] as well as parasite density [38] would increase the precision predicting resistance; in practice, molecular markers need to be simple and easy to apply. Another important factor associated with treatment success is drug quality, whether or not good manufacturing practice standards have been followed. However, treatment efficacy assessment and molecular markers appear to be of limited use when it comes to assessing the usefulness and appropriateness of IPT-SP. Indeed, when used for malaria prevention, SP appears in some settings to function despite high rates of resistant haplotypes and reduced efficacy in treatment of symptomatic malaria.

Gametocyte carriage is known to be elevated following SP failure [39], *dhfr *mutants were associated with increased gametocytaemia despite a high therapeutic efficacy of SP in Colombia [40]. Gametocyte carriage one week following treatment was increased in our study, contemporaneous with the high frequency of *dhfr *triple mutant parasites. These findings lead to the assumption that *dhfr *mutations might influence gametocyte prevalence.

## Conclusions

These results show the high prevalence of *dhfr *triple mutant and some *dhps *point mutations in Gabon, which could explain the longer parasite clearance time, increased SP treatment failure rates and the high rate of gametocyte carriers after SP treatment.

This paper supports the appropriateness of discouraging the use of SP for the treatment of malaria in sub-Saharan Africa as reflected in current guidelines with a shift towards artemisinin-based combination therapy; hence, SP drug pressure is likely to decrease soon, which may limit the spread of SP-resistant parasites and therefore permit SP to continue to be used for IPT in pregnant women and infants whenever applicable.

What are the implications for IPTi-SP as a possible tool for malaria control in the Gabonese setting? The original IPTi trial in Gabon [21] yielded reductions in malaria episodes and anemia in the same order of magnitude as others [14], yet did not reach statistical significance due to a variety of reasons, including a pronounced Healthy Cohort Effect. The current World Health Organization guideline on IPTi with SP in children [15] recommends a ≥ 50% cut-off of *dhps *540E as benchmark for discouraging IPTi-SP use. In the particular setting of the study, the results show for the first time isolates carrying this polymorphism in a small sample size, and in correlation with significant treatment failure. In summary, molecular markers of SP resistance and particularly *dhfr *triple mutants and *dhps *540E need to be closely monitored in Gabon. Although the *dhps *540E benchmark of 50% has not been formally reached in this study, Gabon is at present most likely to be considered as one of those countries falling below the threshold in the decision-making for IPTi-SP introduction.

## Competing interests

The authors declare that they have no competing interests.

## Authors' contributions

GMN, SO, CR, PGK and MPG conceived the paper and designed the studies reported. GMN performed the molecular analyses. RO, JFJK and CR contributed to the molecular data analysis. SO, JJG, KCG, KP, BG, FK, BL, SI, MPG contributed to the clinical field work. GMN, SO and MPG drafted the paper. All authors have contributed to the writing, and approved of the final version of the paper.

## References

[B1] Greenwood BM Bojang K Whitty CJM Targett GAT Malaria Lancet 2005 365 1487 1498 10.1016/S0140-6736(05)66420-3 15850634

[B2] Cowman AF Morry MJ Biggs BA Cross GA Foote SJ Amino acid changes linked to pyrimethamine resistance in the dihydrofolate reductasethymidylate synthase gene of *Plasmodium falciparum* Proc Natl Acad Sci USA 1988 85 9109 9113 10.1073/pnas.85.23.9109 3057499 PMC282673

[B3] Peterson DS Walliker D Wellems TE Evidence that a point mutation in dihydrofolate reductase-thymidylate synthase confers resistance to pyrimethamine in falciparum malaria Proc Natl Acad Sci USA 1988 85 9114 9118 10.1073/pnas.85.23.9114 2904149 PMC282674

[B4] Brooks DR Wang P Read M Watkins WM Sims PF Hyde JE Sequence variation of the hydroxymethyldihydropterin pyrophosphokinase: Dihydropteroate synthase gene in lines of the human malaria parasite, *Plasmodium falciparum*, with differing resistance to sulfadoxine Eur J Biochem 1994 224 397 405 10.1111/j.1432-1033.1994.00397.x 7925353

[B5] Triglia T Cowman AF Primary structure and expression of the dihydropteroate synthetase gene of *Plasmodium falciparum* Proc Natl Acad Sci USA 1994 91 7149 7153 10.1073/pnas.91.15.7149 8041761 PMC44356

[B6] Roper C Pearce R Bredenkamp B Gumede J Drakeley C Mosha F Chandramohan D Sharp B Antifolate antimalarial resistance in southeast Africa: A population-based analysis Lancet 2003 361 1174 1181 10.1016/S0140-6736(03)12951-0 12686039

[B7] Schellenberg D Kahigwa E Drakeley C Malenda A Wigayi J Msokame C Aponte JJ Tanner M Mshinda H Menendez C Alonso PL The safety and efficacy of sulfadoxine-pyrimethamine, amodiaquine and their combination in the treatment of uncomplicated *Plasmodium falciparum *malaria Am J Trop Med Hyg 2002 67 17 23 12363058 10.4269/ajtmh.2002.67.17

[B8] Mockenhaupt FP Ehrhardt S Dzisi SY Teun Bousema J Wassilew N Schreiber J Anemana SD Cramer Jakob P Otchwemah RN Sauerwein RW Eggelte TA Bienzle U A randomised, placebo-controlled, and double-blind trial on sulfadoxinepyrimethamine alone or combined with artesunate or amodiaquine in uncomplicated malaria Trop Med Int Health 2005 10 512 520 10.1111/j.1365-3156.2005.01427.x 15941413

[B9] Abacassamo F Enosse S Aponte JJ Gómez-Olive FX Quintó L Mabunda S Barreto A Magnussen P Ronn AM Thompson R Alonso PL Efficacy of chloroquine, amodiaquine, sulphadoxine-pyrimethamine and combination therapy with artesunate in Mozambiquan children with noncomplicated malaria Trop Med Int Health 2004 9 200 208 10.1046/j.1365-3156.2003.01182.x 15040556

[B10] Alloueche A Bailey W Barton S Bwika J Chimpeni P Falade CO Fehintola FA Horton J Jaffar S Kanyok T Kremsner PG Kublin JG Lang T Missinou MA Mkandala C Oduola AM Premji Z Robertson L Sowunmi A Ward SA Winstanley PA Comparison of chlorproguanil-dapsone with sulfadoxine-pyrimethamine for the treatment of uncomplicated falciparum malaria in young African children: double-blind randomised controlled trial Lancet 2004 363 1843 1848 10.1016/S0140-6736(04)16350-2 15183620

[B11] Oduro AR Anyorigiya T Hodgson A Ansah P Anto F Ansah NA Atuguba F Mumuni G Amankwa J A randomized comparative study of chloroquine, amodiaquine and sulphadoxine-pyrimethamine for the treatment of uncomplicated malaria in Ghana Trop Med Int Health 2005 10 279 284 10.1111/j.1365-3156.2004.01382.x 15730512

[B12] Kremsner PG Krishna A Antimalarial combinations Lancet 2004 364 285 294 10.1016/S0140-6736(04)16680-4 15262108

[B13] Grobusch MP Egan A Gosling RD Newman RD Intermittent preventive therapy for malaria: Progress and future directions Curr Opin Infect Dis 2007 20 613 620 10.1097/QCO.0b013e3282f1ae3b 17975412

[B14] Aponte JJ Schellenberg D Egan A Breckenridge A Carneiro I Critchley J Danquah I Dodoo A Kobbe R Lell B May J Premji Z Sanz S Sevene E Soulaymani-Becheikh R Winstanley P Adjei S Anemana S Chandramohan D Issifou S Mockenhaupt F Owusu-Agyei S Greenwood B Grobusch MP Kremsner PG Macete E Mshinda H Newman RD Slutsker L Tanner M Alonso P Menendez C Intermittent Preventive Treatment for malaria control in African Infants: Pooled analysis of safety and efficacy in six randomized controlled trials Lancet 2009 374 1533 1542 10.1016/S0140-6736(09)61258-7 19765816

[B15] WHO Policy recommendation on Intermittent Preventive Treatment during infancy with sulfadoxine-pyrimethamine (SP-IPTi) for Plasmodium falciparum malaria control in Africa 2010 World Health Organization, Geneva https://who.int/malaria/news/WHO_policy_recommendation_IPT_032010.pdf [last accessed on 2 December 2010]

[B16] Wernsdorfer WH Noedl H Molecular markers for drug resistance in malaria: use in treatment, diagnosis and epidemiology Curr Opin Infect Dis 2003 16 553 558 10.1097/00001432-200312000-00007 14624105

[B17] Pearce RJ Pota H Evehe MS Bâel H Mombo-Ngoma G Malisa AL Ord R Inojosa W Matondo A Diallo DA Mbacham W van den Broek IV Swarthout TD Getachew A Dejene A Grobusch MP Njie F Dunyo S Kweku M Owusu-Agye S Chandramohan D Bonnet M Guthmann JP Clarke S Barnes KL Streat E Katokele ST Uusiku P Agboghoroma CO Elegba OY Cisse B A-Elbasit IE Giha HA Kachur SP Lynch C Rwakimari JB Chanda P Hawela M Sharp B Naidoo I Roper R Multiple origins and regional dispersal of resistant *dhps *in Afrcian *Plasmodium falciparum *malaria PLoS Med 2009 6 e1000055 10.1371/journal.pmed.1000055 19365539 PMC2661256

[B18] Bwijo B Kaneko A Takechi M Zungu IL Moriyama Y Lum JK Tsukahara T Mita T Takahashi N Bergqvist Y Björkman A Kobayakawa T High prevalence of quintuple mutant *dhps/dhfr *genes in *Plasmodium falciparum *infections seven years after introduction of sulfadoxine and pyrimethamine as first line treatment in Malawi Acta Trop 2003 85 363 373 10.1016/S0001-706X(02)00264-4 12659974

[B19] Plowe CV Cortese JF Djimde A Nwanyanwu OC Watkins WM Winstanley PA Estrada-Franco JG Mollinedo RE Avila JC Cespedes JL Carter D Doumbo OK Mutations in *Plasmodium falciparum *dihydrofolate reductase and dihydropteroate synthase and epidemiologic patterns of pyrimethamine-sulfadoxine use and resistance J Infect Dis 1997 176 1590 1596 10.1086/514159 9395372

[B20] Ramharter M Adegnika AA Agnandji ST Matsiegui PB Grobusch MP Winkler S Graninger W Krishna J Yazdanbakhsh M Mordmueller B Lell B Missinou MA Mavoungou E Issifou S Kremsner PG History and perspectives of medical research at the Albert Schweitzer Hospital in Lambaréné, Gabon Wien Klin Wochenschr 2007 11 Suppl 3 8 12 10.1007/s00508-007-0857-5 17987353

[B21] Grobusch MP Lell B Schwarz NG Gabor J Dörnemann J Pötschke M Oyakhirome S Kiessling GC Necek M Langin MU Klouwenberg PK Klopfer A Naumann B Altun H Agnandji ST Goesch J Decker M Salazar CL Supan C Kombila DU Borchert L Koster KB Pongratz P Adegnika AA Glasenapp I Issifou S Kremsner PG Intermittent preventive treatment against malaria in infants in Gabon--a randomized, double-blind, placebo-controlled trial J Infect Dis 2007 196 1595 1602 10.1086/522160 18008242

[B22] Grobusch MP Gabor JJ Aponte JJ Schwarz NG Poetschke M Doernemann J Schuster K Koester KB Profanter K Borchert LB Kurth F Pongratz P Issifou S Lell B Kremsner PG No rebound of morbidity following intermittent preventive sulfadoxine-pyrimethamine treatment of malaria in infants in Gabon J Infect Dis 2009 200 1658 1661 10.1086/647990 19848610

[B23] Classification of treatment outcome WHO 2005 http://www.malariadrugresistance.net/warn/clinical/files/WHOClassificationofTreatmentOutcomes.pdf accessed 12 February, 2007

[B24] Pearce RJ Drakeley C Chandramohan D Mosha F Roper C Molecular determination of point mutation haplotypes in the dihydrofolate reductase and dihydropteroate synthase of *Plasmodium falciparum *in three districts of northern Tanzania Antimicrob Agents Chemother 2003 47 1347 1354 10.1128/AAC.47.4.1347-1354.2003 12654669 PMC152520

[B25] Cattamanchi A Kyabayinze D Hubbard A Rosenthal PJ Dorsey G Distinguishing recrudescence from reinfection in a longitudinal antimalarial drug efficacy study: comparison of results based on genotyping of msp-1, msp-2, and glurp Am J Trop Med Hyg 2003 68 133 139 12641400

[B26] Planche T Krishna S Kombila M Engel K Faucher JF Ngou-Milama E Kremsner PG Comparison of methods for the rapid laboratory assessment of children with malaria Am J Trop Med Hyg 2001 65 599 602 11716121 10.4269/ajtmh.2001.65.599

[B27] Oyakhirome S Issifou S Pongratz P Barondi F Ramharter M Kun JFJ Missinou MA Lell B Kremsner PG Fosmidomycin-clindamycin versus sulfadoxine-pyrimethamine in the treatment of *Plasmodium falciparum *malaria: A randomized controlled trial Antimicrob Agents Chemother 2007 51 1869 1871 10.1128/AAC.01448-06 17325227 PMC1855537

[B28] Kun FJK Lehman LG Lell B Schmidt-Ott R Kremsner PG Low-dose treatment with sulfadoxine -pyrimethamine combinations selects for drug-resistant *Plasmodium falciparum *strains Antimicrob Agents Chemother 1999 43 2205 2208 10471565 10.1128/aac.43.9.2205 PMC89447

[B29] Mawili-Mboumba DP Ekala MT Lekoulou F Ntoumi F Molecular analysis of DHFR and DHPS genes in *P. falciparum *clinical isolates from the Haut-Ogooué region in Gabon Acta Trop 2001 78 231 240 10.1016/S0001-706X(01)00084-5 11311186

[B30] Aubouy A Jafari S Huart V Migot-Nabias F Mayombo J Durand R Bakary M Le Bras J Deloron P *DHFR *and *DHPS *genotypes of *Plasmodium falciparum *isolates from Gabon correlate with in vitro activity of pyrimethamine and cycloguanil, but not with sulfadoxine-pyrimethamine treatment efficacy J Antimicrob Chemother 2003 52 43 49 10.1093/jac/dkg294 12805261

[B31] Metzger W Mordmüller B Graninger W Bienzle U Kremsner PG Sulfadoxine/pyrimethamine or chloroquine/clindamycin treatment of Gabonese school children infected with chloroquine resistant malaria J Antimicr Chemother 1995 36 723 728 10.1093/jac/36.4.723 8591949

[B32] Lell B Lehman LG Schmidt-Ott JR Sturchler D Handschin J Kremsner PG Malaria chemotherapy trial at a minimal effective dose of mefloquine/sulfadoxine-pyrimethamine compared with equivalent doses of sulfadoxine-pyrimethamine or mefloquine alone Am J TropMed Hyg 1998 58 619 624 10.4269/ajtmh.1998.58.619 9598451

[B33] Deloron P Mayombo J Le Cardinal A Mezui-Me-Ndong J Bruzi-Baert C Lekoulou F Elissa N Sulfadoxine-pyrimethamine for the treatment of *Plasmodium falciparum *malaria in Gabonese children Trans R Soc Trop Med Hyg 2000 94 188 190 10.1016/S0035-9203(00)90272-4 10897366

[B34] Nsimba B Guiyedi V Mabika-Mamfoumbi M Mourou-Mbina JR Ngoungou E Bouyou-Akotet M Loembet R Durand R Le Bras J Kombila M Sulphadoxine/pyrimethamine versus amodiaquine for treating uncomplicated childhood malaria in Gabon: A randomized trial to guide national policy Malar J 2008 7 31 10.1186/1475-2875-7-31 18267042 PMC2276509

[B35] Winkler S Brandts C Wernsdorfer WH Graninger W Bienzle U Kremsner PG Drug sensitivity of *Plasmodium falciparum *in Gabon. Activity correlations between various antimalarials Trop Med Parasitol 1994 45 214 218 7899790

[B36] Van Hensbroek MB Morris-Jones S Meisner S Jaffar S Bayo L Dackour R Phillips C Greenwood BM Iron, but not folic acid, combined with effective antimalarial therapy promotes haematological recovery in African children after acute falciparum malaria Trans R Soc Trop Med Hyg 1995 89 672 676 10.1016/0035-9203(95)90438-7 8594693

[B37] Dzinjalamala FK Macheso A Kublin JG Taylor TE Barnes KI Molyneux ME Plowe CV Smith PJ Blood folate concentrations and in vivo sulfadoxine-pyrimethamine failure in Malawian children with uncomplicated *Plasmodium falciparum *malaria Am J Trop Med Hyg 2005 72 267 272 15772319

[B38] Ehrhardt S Mockenhaupt FP Agana-Nsiire P Mathieu A Anemana SD Stark K Otchwemah RN Bienzle U Efficacy of chloroquine in the treatment of uncomplicated *Plasmodium falciparum *malaria in northern Ghana Ann Trop Med Parasitol 2002 96 239 247 10.1179/000349802125000772 12061971

[B39] Bousema JT Gouagna LC Meutstege AM Okech BE Akim NI Githure JL Beier JC Sauerwein RW Treatment failure of pyrimethamine-sulphadoxine and induction of *Plasmodium falciparum *gametocytaemia in children in western Kenya Trop Med Int Health 2003 8 427 430 10.1046/j.1365-3156.2003.01047.x 12753638

[B40] Méndez F Munoz A Carrasquilla G Jurado D Arévalo-Herrera M Cortese JF Plowe CV Determinants of treatment response to sulfadoxine-pyrimethamine and subsequent transmission potential in falciparum malaria Am J Epidem 2002 156 230 238 10.1093/aje/kwf030 12142257

